# Development and validation of a paediatric long-bone fracture classification. A prospective multicentre study in 13 European paediatric trauma centres

**DOI:** 10.1186/1471-2474-12-89

**Published:** 2011-05-06

**Authors:** Dorien Schneidmüller, Christoph Röder, Ralf Kraus, Ingo Marzi, Martin Kaiser, Daniel Dietrich, Lutz von Laer

**Affiliations:** 1 Department of Trauma, Hand and Reconstructive Surgery, Hospital of the J.W. Goethe-University of Frankfurt, Theodor-Stern-Kai 7, 60590 Frankfurt am Main, Germany; 2 MEM Research Center, University of Bern, Institute for Evaluative Research in Orthopaedic Surgery, Stauffacherstr. 78, 3014 Bern, Switzerland; 3 Department of Trauma, Hand and Reconstructive Surgery, Hospital of the Justus Liebig University of Giessen, Rudolf-Buchheim-Str. 8, 35392 Giessen, Germany; 4 Department of paediatric surgery, Hospital of the University of Schleswig-Holstein, Ratzeburger Allee 160, 23538 Lübeck, Germany; 5 Institute for Mathematical Statistics and Actuarial Science, University of Bern, Sidlerstrasse 5, 3012 Bern, Switzerland; 6 Prof. Emeritus, Department of Paediatric Surgery, Hospital of the University of Basle, Spitalstrasse 33, 4056 Basel, Switzerland

## Abstract

**Background:**

The aim of this study was to develop a child-specific classification system for long bone fractures and to examine its reliability and validity on the basis of a prospective multicentre study.

**Methods:**

Using the sequentially developed classification system, three samples of between 30 and 185 paediatric limb fractures from a pool of 2308 fractures documented in two multicenter studies were analysed in a blinded fashion by eight orthopaedic surgeons, on a total of 5 occasions. Intra- and interobserver reliability and accuracy were calculated.

**Results:**

The reliability improved with successive simplification of the classification. The final version resulted in an overall interobserver agreement of κ = 0.71 with no significant difference between experienced and less experienced raters.

**Conclusions:**

In conclusion, the evaluation of the newly proposed classification system resulted in a reliable and routinely applicable system, for which training in its proper use may further improve the reliability. It can be recommended as a useful tool for clinical practice and offers the option for developing treatment recommendations and outcome predictions in the future.

## Background

Classification systems are widely used in orthopaedic and trauma surgery. They play a key role in the reporting of clinical and epidemiological data, allowing for uniform comparison and documentation of different conditions. They constitute the semantic basis of retrospective and prospective clinical studies by providing a common language for defining and categorising pathology. This is becoming increasingly important in the implementation of quality control measures for diagnostic and therapeutic procedures. Therefore, a feasible and standardised form of documentation is required that is accessible for everyone and easy to use.

A useful classification system must be reliable and accurate before it can be considered valid [1,2]. Reliability reflects the precision of a classification system and in general refers to intraobserver and interobserver reliability. The intraobserver reliability describes the agreement between the ratings of one observer performing repeated classifications of a given entity, whereas the interobserver reliability describes the agreement between the ratings of different observers. Most of the classification studies use the Kappa coefficient introduced by Cohen [3] to quantify the agreement between raters. It distinguishes true agreement between various observations from agreement due to chance alone, and is expressed as a value between -1 and 1. A Kappa value of -1.0 means complete disagreement, 0.0 means chance agreement and 1.0, complete agreement. Different criteria are given in the literature for assessing the strength of agreement. The most widely adopted are those of Landis and Koch [4].

Classification accuracy is described using latent class modelling. The hypothesis is that each fracture belongs to one of several real clinically relevant classes, which may be theoretically defined, but not directly observable in practice. These classes are said to be "latent". The analysis aims to identify the most likely number of these latent classes in the population, given the selected sample of fractures and the agreement data collected among the various raters. For each class, the accuracy of classification by each rater is estimated [5,6].

Numerous fracture classification systems have been proposed in orthopaedics [7-23]. Specific paediatric classifications are less common. It does not seem appropriate to adopt a classification system created for adults for use in paediatric orthopaedics because certain child-specific factors must be considered. The growing bone has the capability of spontaneous corrections of remaining deviations as well as the risk of growth disturbances. To date, only one child specific classification system for long bone fractures has been published [24,25].

The aim of this study was to develop a specific classification system for paediatric long bone fractures together with a digital documentation system. The classification is based on a preliminary version published in 2000 [26-28], which has been further developed, improved and evaluated with respect to intraobserver and interobserver reliability and accuracy.

## Methods

In the years 2003 and 2005 two prospective multicentre studies documenting a total of 2308 fractures were conducted in 13 paediatric trauma centres in Germany, Switzerland and Austria. All participants were active members of the Li-La paediatric expert group [29-31]. In each study hospital, all consecutively treated long bone fractures in children up to and including 16 years of age were assessed over a period of 3 months. The institutional review boards of the Universities of Bern, Switzerland, and Giessen, Germany had approved the project.

Demographic data such as sex and age, history and important clinical findings were collected with the MEMdoc documentation portal of the Institute for Evaluative Research in Medicine of the University of Bern, Switzerland [32]. Primary and follow up x-rays were scanned, uploaded via the MEMdoc web interface and centrally stored with every patient record. To limit selection bias, all cases were included even if the quality of diagnostic images was not perfect.

On the basis of the frequency distribution of fracture types in the data-set, 30 x-rays representing the most common fracture types were extracted from the pool of 2308 for use in a pilot study. Typical radiographs were selected by 2 orthopaedic surgeons who were not assessors in the study. These fractures were assessed using the new classification system. Eight observers with different levels of experience participated: three consultant surgeons specialised in paediatric trauma and five orthopaedic residents. All raters were blinded to any information about the patient. The patient identification and the date on the films were hidden and each case was identified with a random number only. In a common rating session this series of 30 x-rays was studied and evaluated individually by each practitioner.

On the basis of these results a sample size calculation was performed and an expanded group comprising 150 cases (including the initial 30 pilot cases) was created from the pool of 2308 fractures in order to cover the complete spectrum of fracture types. These 150 cases were classified by the same observers, 6 months after the initial series of 30 cases. This allowed the evaluation of the inter- and intraobserver reliability in relation to the initial 30 cases.

Following analysis of the results, a simplification of the classification system was introduced. This was evaluated by the same observers again rating the same 150 cases, randomly presented to them, after a further interval of 6 months.

**Figure 1:**
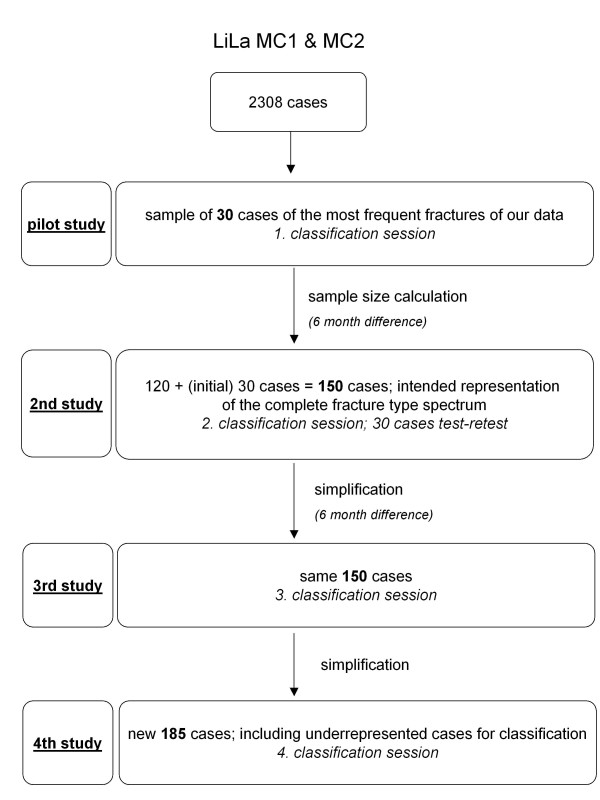
**Flow chart of study history**.

For the last agreement study, a completely new fracture sample was selected that also included more cases of some previously underrepresented fracture types for which the classification system had been revised again. In this way, a new set of 185 fractures was compiled (Figure 1).

In summary the development and validation process included a series of four formal agreement studies intended to allow for continual improvement of the classification system by reviewing the results, identifying specific flaws and subsequently adjusting the coding.

### Statistical analysis

For the first classification session, sample size estimation was performed based on the 30 cases from the 2003 multicentre study. These 30 cases were classified again by all 8 raters as part of the first classification session with the total 150 fractures. The interobserver reliability for those 30 cases was estimated using Kappa coefficients to indicate the degree of agreement in ratings [33]. The last classification session was conducted with 185 selected cases to guarantee a sufficient number of examples of the most important fracture types. The analyses were performed for all raters stratified by experience (senior and resident level). For the first letter of the classification code (Classification Dimension; CD1) all cases were used, for the second one (CD2) only the cases with agreement on CD1, for CD3 the cases with agreement on CD1 and CD2 etc. Calculations were done with the MAGREE macro of SAS (SAS Institute Inc., Cary, NC, USA).

A gold standard was predefined by consensus amongst two independent senior surgeons. It was used for classification accuracy for each category by each rater (percentage of cases correctly classified) and checked by "Latent Class Modelling" using the software latent GOLD^® ^(Statistical Innovations Inc. Belmont, MA, USA).

Over a timeframe of 6 months, two raters classified the final 185 fractures twice. For each of the two raters the percent agreement between the first and the second ratings and the intraobserver Kappa coefficient were calculated. This was done for CD1, CD1-2, CD1-3 and CD1-4. The mean agreement and mean kappa values for the two raters were calculated.

### Classification system

**Figure 2:**
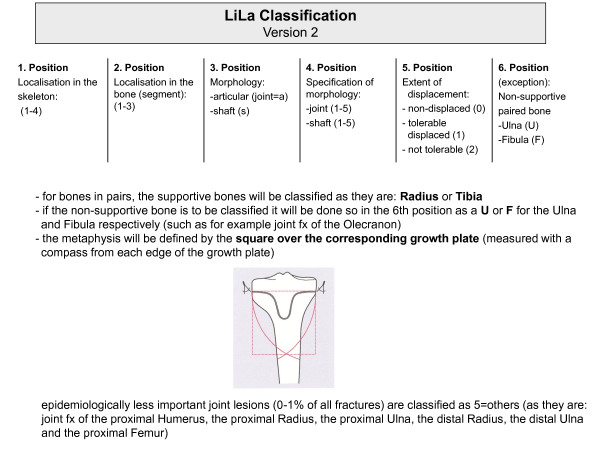
**Overall structure of the Li-La Classification of paediatric fractures of long bones**.

The final classification code consists of five (optionally six) digits (Figure 2):

1. According to the AO classification of long bone fractures in adults [34] the first digit represents the affected part of the upper or lower extremity:

• 1 = humerus

• 2 = forearm

• 3 = femur

• 4 = lower leg

2. The second digit represents the bone segment where the fracture is located:

• 1 = proximal (including epiphysis and metaphysis)

• 2 = middle (diaphysis/shaft)

• 3 = distal (including epiphysis and metaphysis).

The metaphysis is defined by a square over the growth plate of the affected bone (Figure 2).

3. Because of its therapeutic relevance the third digit indicates the assessor's decision as to whether it is an articular or non-articular (shaft) fracture.

• All fractures affecting the articular surface, be it the epiphysis or the metaphysis (fractures of the olecranon), are considered to be articular;(a).

• All fractures of the shaft and metaphysis are considered to be non-articular: (s).

4. The fourth digit specificies the morphology of the fracture type for articular and shaft fractures separately.

• Articular fractures:

• 1 = epiphyseal with wide open physis (Salter III)

• 2 = epi-metaphyseal with wide open physis (Salter IV)

• 3 = epiphyseal with beginning physiological closure of the plate in adolescents (two-plane/Tilleaux fracture)

• 4 = epi-metaphyseal with beginning physiological closure of the plate in adolescents (tri-plane fracture).

• 5 = statistically less important joint lesions are subsumed as 5 = others; e.g. intraarticular ligament avulsions and flake fractures.

• Non-articular/shaft fractures:

• 1 = they start with the most peripherical metaphyseal fracture; the epiphyseal separation with or without metaphyseal wedge (Salter I and II)

• 2 = metaphyseal greenstick or buckle fractures and greenstick or bowing fractures of the shaft

• 3 = all complete fractures including transverse, oblique and torsion fractures

• 4 = multifragment fractures

• 5 = statistically less important shaft lesions are subsumed as 5 = others; e.g. extra-articular ligament avulsions.

5. A fifth optional digit was introduced to divide the fracture displacement into

• 0 = non-displaced

• 1 = tolerable displacement

• 2 = intolerable displacement

to indicate the likelihood of spontaneous correction of displacement by further growth. Tolerable displacement indicates displacement that is reliably known to either correct itself spontaneously during further growth or, in case it persists, to have no clinically relevant functional or cosmetic consequences. To date this is still an individual, subjective decision. Provisionally, a fracture gap greater than 2 mm is considered to represent displacement in all epiphyseal fractures[35].

6. The sixth digit helps to specify the fractures of paired bones (forearm and lower leg). In general, the supportive bone is classified as it is: Radius for the forearm and Tibia for the lower leg. If the other bone is affected and needs special description, for example with a fracture of the proximal Ulna, isolated fracture of the ulna or fibula, **U **will be used for ulna and **F **for fibula.

There is only one exception to this classification pattern. Because of their frequency and peculiarities in fracture healing and possible complications, fractures of the distal humerus received a separate designation

• 1 = fracture of the radial condyle

• 2 = Y-fracture

• 3 = fracture of the ulnar condyle

**Figure 3:**
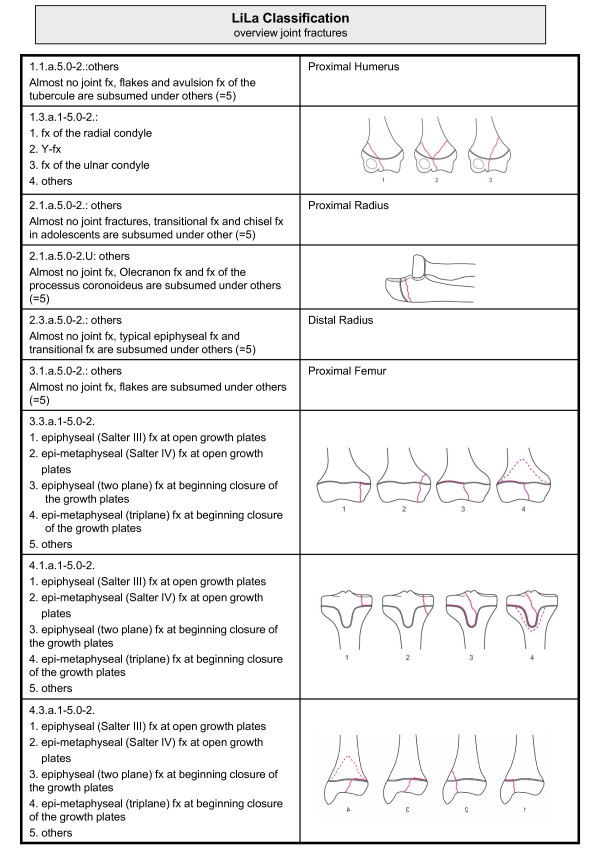
**Overview of Li-La classification of paediatric long bone fractures: articular fractures**.

**Figure 4:**
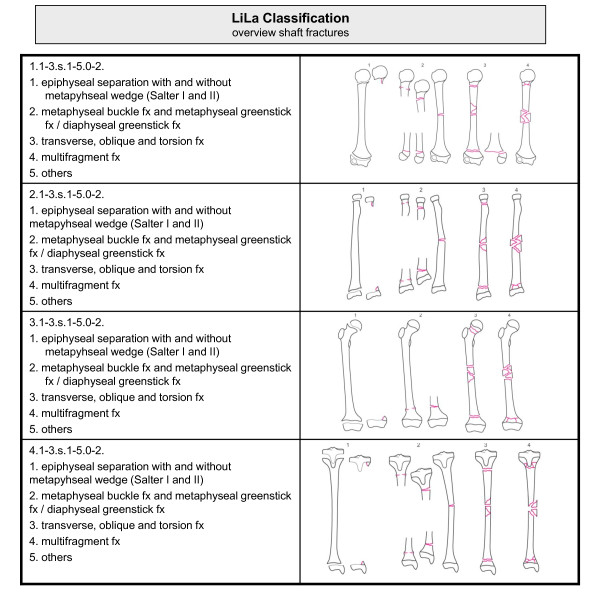
**Overview of Li-La classification of paediatric long bone fractures: shaft fractures**.

**Figure 5:**
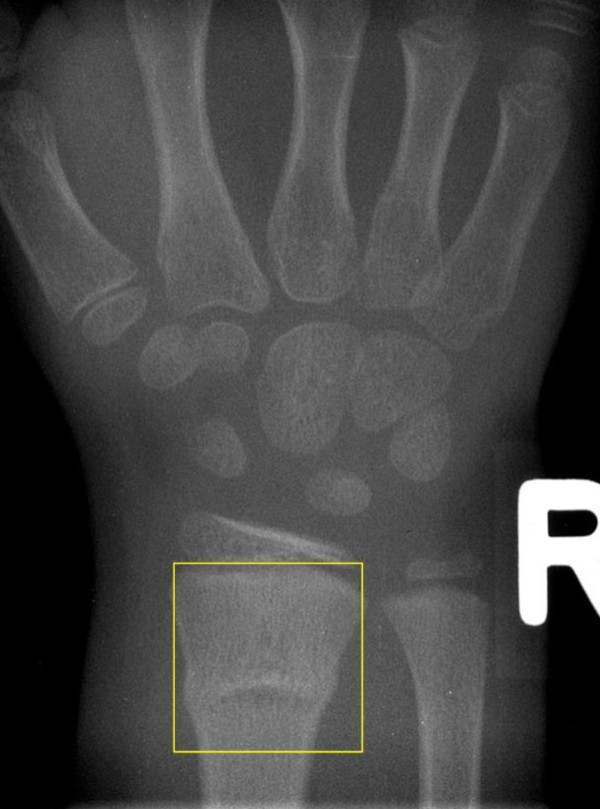
**A buckle fracture of the distal radius**. The classification is determined as follows: localization in the skeleton - radius = 2; localization in the bone - metaphysis (square rule) = 3; morphology - shaft = s; fracture type - buckle fracture = 2; displacement - non-displaced = 0. Code: 2.3.s.2.0.

An overview of the classification system is given in Figures 3 and 4. An example is provided in Figure 5.

## Results

The overall case pool that was included in the development of the classification system comprised 2308 fractures. Male patients were slightly overrepresented with 56.8%. The risk of having a fracture before termination of growth was 1.2-1.6-fold higher in males. The average overall age of the patients was 8.1 years. The main localisation of fracture was the forearm (54.1%), followed by the humerus (20.3%), the lower leg (20.4%) and the femur (5.2%). 2/3 of all fractures involved the metaphysis (65.1%), whereas fractures of the diaphysis occurred in 24.8% and fractures of the epiphysis in 8.1% of all cases. Most fractures occurred as a result of sports-related injuries (38.5%), followed by domestic accidents (23.0%) and playground accidents (19.9%) [30].

### Intraobserver agreements

Intraobserver agreement was determined with the 30 cases used for sample size calculation in the very first agreement study and with the 185 cases of the final study. In the first series, there was test-retest agreement in 96% of cases for the first two dimensions, in 91.4% of cases for the first three dimensions, in 89.1% of cases for the first four dimensions, in 74.7% of cases for the first five dimensions and in 19.6% of cases for all six dimensions. This equated to Kappa values ranging from 0.97 to 0.57. In the final version there was test-retest agreement in 97% of cases for the first two dimensions, in 97% of cases for the first three dimensions, and in 87% of cases for the first four dimensions. This equated to Kappa values ranging from 0.99 to 0.86.

### Interobserver agreement

The overall interobserver reliability of the initial classification was κ = 0.58. Different Kappa values were found for the single dimensions. Assessing the localisation in the skeleton (CD1) and the paired bone (CD 6) showed the best agreement (localisation in skeleton κ = 0.99, localisation in bone κ = 0.91 and paired bone κ = 0.99), whereas there was less agreement in assigning the child-specific fracture code (CD 4) with κ = 0.66. Classification of the segment (CD 2 - metaphysis, epiphysis, diaphysis) showed only weak agreement κ = 0.33.

The only moderate agreement in the initial version was largely explained by the difficulty in distinguishing the metaphysis from the diaphysis, the greenstick from the buckle fracture and the transverse from the oblique diaphyseal fracture. Due to a lack of therapeutic relevance, e.g. their requirement for similar or identical treatment, some fracture types (e.g. metaphyseal greenstick and buckle fracture) were subsumed in one group and the square over the physis was introduced to differentiate the distal part from the middle, i.e. the shaft. Indeed, this simplification resulted in an improvement in the agreement in ratings for the subsequent version of the classification system. Results for each dimension are based on all cases with agreement in the preceding dimension. Those cases with disagreement in the preceding dimension were not considered.

After analysing the problems with the initial version in the first 3 series, the classification system was modified in the final series and then re-evaluated.

• Dimension 1: no change was made

• Dimension 2 (localisation in bone: segment): assigning the fracture localisation in the bone to a distal or proximal part, including the epiphysis and metaphysis, and a diaphysial part by defining the metaphysis with a square over the physis improved agreement from қ = 0.33 in the initial version to қ = 0.89 in the final version (177 of 185 cases applicable; Table 1).

**Table 1:** Summary of agreement und Kappa values for CD 2 = localisation in the bone

CD2	1	2	3	all
Agreement	17	39	85	141
Total cases	20	49	108	177
Kappa all	0.95	0.86	0.90	**0.89**
Kappa senior	0.95	0.92	0.96	0.94
Kappa junior	0.95	0.84	0.87	0.87

^1^proximal segment, ^2^diaphysis, ^3^distal segment

• Dimension 3 (morphology): distribution of fractures according to articular involvement. The overall Kappa coefficient was қ = 0.88 (141 of 185 cases applicable; Table 2). The accuracy of classification of articular and shaft fractures for the multicenter study are shown in Table 3.

**Table 2:** Summary of agreement und Kappa values for CD 3 = morphology

CD3	A	S	all
Agreement	11	116	125
Total cases	18	123	141
Kappa all	0.90	0.89	**0.88**
Kappa senior	0.98	0.96	0.96
Kappa junior	0.85	0.85	0.84
Full rater agreement (latent class)	88.7%		

^a^articular, ^s^non-articular/shaft

**Table 3:** Accuracy of classification of articular and shaft fractures (A/S).

Likely fracture type	A (n = 18)	S (n = 123)
Fracture class distribution	12.8%	87.2%
Rater 1^1^		
A	**94.4%**	0%
S	5.6%	**100%**
Rater 2^1^		
A	**100%**	0%
S	0%	**100%**
Rater 3^1^		
A	**100%**	0%
S	0%	**100%**
Rater 4^2^		
A	**94.4%**	0%
S	5.6%	**100%**
Rater 5^2^		
A	**94.4%**	0.8%
S	5.6%	**99.2%**
Rater 6^2^		
A	**88.9%**	3.3%
S	11.1%	**96.7%**
Rater 7^2^		
A	**83.3%**	0%
S	16.7%	**100%**
Rater 8^2^		
A	**100%**	0%
S	0%	**100%**

Fracture class distribution and probability of correct classification for each fracture class and rater.

^1^senior rater, ^2^junior rater

• Dimension 4: after subsuming fractures with the same therapeutic consequence in one group, specification of the child-specific morphology of the fracture resulted in a mean Kappa coefficient of қ = 0.72 (127 of 185 cases applicable; Table 4). Agreement separated by fracture type (epiphysis, metaphysis, diaphysis) ranged from қ = 0.59-0.92 in the multicenter study (Table 5).

**Table 4:** Summary of agreement und Kappa values for CD4 = child-specific fracture code

CD4	1	2	3	4	5	all
Agreement	14	25	19	0	3	61
Total cases	22	50	49	2	4	127
Kappa all	0.79	0.70	0.69	0.43	0.94	**0.72**
Kappa senior	0.86	0.67	0.57	0.27	0.91	0.68
Kappa junior	0.76	0.74	0.77	0.54	0.95	0.76

*definition of CD4 outcomes 1-5 depends on articular or non-articular/shaft fracture

**Table 5:** Overall assessment (dimensions CD1 - 4) (127 cases applicable, agreement in dimensions 1-3 must have been achieved)

Localisation in skeleton	Localisation in bone	Articular/shaft	Child specific fracture code	N	Kappa
1,2,3,4	1 and 3 (Epiphysis)	a	1 (Salter I)	8	0.83
			2 (Salter II)	-	-
			3 (Two plane)	-	-
			4 (Triplane)	-	-
			5 (others)	3	0.90
			**All**	**11**	**0.79**
					
1,2,3,4	1 and 3 (Metaphysis)	s	1 (epiphysiolysis)	14	0.72
			2 (incomplete Fx)	40	0.71
			3 (complete Fx)	23	0.64
			4 (multifragment Fx)	-	-
			5 (other)	1	n.a
			**All**	**78**	**0.69**
					
1,2,3,4	2 (Diaphysis)	s	1 non existent		
			2 (incomplete Fx)	10	0.59
			3 (complete Fx)	26	0.60
			4 (multifragment Fx)	2	n.a
			5 (other)	-	-
			**All**	**38**	**0.58**

• Dimension 5 (optional): all fractures were classified according to their subjective prognosis and therapeutic relevance as non-displaced (0), displaced but tolerable (1) and displaced and intolerable (2). Table 6 shows the Kappa coefficients for these. The results were not so favourable with a mean Kappa of қ = 0.61. Subsuming the undisplaced (0) and the tolerable (1) fractures because of lack of therapeutic relevance resulted in a mean Kappa of қ = 0.83 (Table 7) (61 of 185 cases applicable).

**Table 6:** Summary of agreement und Kappa values for CD5 = displacement

CD5	0	1	2	all
Agreement	3	1	21	25
Total cases	16	15	30	61
Kappa all	0.59	0.30	0.83	**0.61**
Kappa senior	0.73	0.24	0.80	0.65
Kappa junior	0.47	0.29	0.83	0.56

^0^not displaced, ^1^displaced but tolerable, ^2^displaced not tolerable

**Table 7:** Summary agreement und Kappa values for CD5 = displacement subsuming the undisplaced and the displaced but tolerable fractures

CD5	0/1	2	all
Agreement	25	21	46
Total cases	31	30	61
Kappa	0.84	0.83	**0.83**
Kappa senior	0.80	0.80	0.80
Kappa junior	0.84	0.83	0.83

^0^not displaced, ^1^displaced but tolerable, ^2^displaced not tolerable

The final version resulted in an overall interobserver agreement of κ = 0.71 for the dimensions CD 1-4. There was no significant difference in κ values between experienced (n = 3, κ = 0.73) and less experienced (n = 5, κ = 0.72) raters. There was perfect agreement between the gold standard and the classification based on latent class modelling for CD1, CD2 and CD3. For CD4 and CD5 there were some minor differences.

## Discussion

Although many classification systems have been widely adopted and frequently used in orthopaedic surgery, few have been scientifically tested for their reliability. Those that *have *been evaluated show generally low reliability but they are nonetheless still in common use.

Considering the differing methodologies used in different studies, it is difficult to interpret the reported Kappa values with confidence. Our results indicated good reliability for dimensions CD 1-4 with an overall Kappa value of 0.71 for a group of clinicians who are interested in the topic; the values were not dependent on surgical experience. The majority of other studies reported lower levels of agreement (Table 8). One exception is the assessment of supracondylar fractures of the distal humerus using a modified Gartland classification [36], which showed an interobserver reliability of κ = 0.74 and an intraobserver reliability of κ = 0.81-0.84. Similarly, an assessment of tibial plateau fractures according to the Schatzker classification, and based on conventional x-rays and MRI scans, revealed an interobserver agreement of κ = 0.85 [23]. The AO paediatric classification shows Kappa coefficients for diagnosis of specific child patterns of 0.51, 0.63, and 0.48 for epiphyseal, metaphyseal, and diaphyseal fractures, respectively. The moderate Kappa values in our initial studies were largely explained by the difficulty in distinguishing the metaphysis from the diaphysis, the greenstick from the buckle fracture and the tranverse from the oblique diaphyseal fracture. As explained earlier, this classification was simplified because of its lack of therapeutic relevance. The metaphyseal buckle and greenstick fractures of the distal radius, for example, require exactly the same treatment, namely cast immobilisation [37]. Thus, discrimination between these two metapyhseal fracture types is not relevant and the simplification resulted in an improvement in the Kappa values for the interobserver reliability.

**Table 8:** Examples of reliability of classification systems

Author	Classification	Interobserver κ	Intraobserver κ
**Humerus**			
Sidor et al. 1993 [33]	Neer (prox humerus)	0.48 + 0.52	0.66
Siebenrock, Gerber 1993 [34]	Neer (prox. humerus)	0.40	0.60
	AO/ASIF	0.53 (types)	0.58 (types)
		0.42 (groups)	0.48 (groups)
			
Sjöden et al. 1999 [35]	Müller AO (prox. humerus)	0.32-0.34	0.29-0.74
	Neer	0.44-0.49	0.27-0.73
Barton et al. 2001 [6]	mod. Gartland (paediatric fracture of the dist. humerus)	0.74	0.81-0.84
			
**Radius**			
Kreder et al. 1996 [19]	Müller AO (dist. radius)	0.33-0.68	0.25-0.42
Illarramendi et al. 1998 [16]	Müller AO (dist. radius)	0.37	0.57
	Frykman	0.43	0.61
Flinkkilä et al. 1998 [14]	Müller AO (dist. radius)	0.18 (x-ray)	-
		0.16 (x-ray + CT)	
			
**Femur**			
Andersen et al. 1990 [2]	Evans (prox. femur)	0.38-0.69	0.56-0.67
Pervez et al. 2002 [28]	Jensen (prox Femur)	0.34	0.34
	AO	0.33	0.42
		0.62 (without subgroups)	0.71 (without subgroups)
Bjørgul, Reikeras 2002 [8]	Garden (femoral neck)	0.4	-
Schipper et al. 2001 [30]	AO/ASIF (prox. femur)	0.33-0.34	0.48
			
**Lower leg**			
Thomsen et al. 1991 [40]	Lauge-Hansen (dist. tibia)	0.49 + 0.60	0.60-0.70
	Weber	0.58 + 0.56	0.60-0.76
Martin et al. 1997 [23]	Rüedi und Allgöwer (dist tibia)	0.46	0.55
	AO/ASIF	0.60 (types)	0.70 (types)
		0.38 (groups)	0.48 (groups)
Swiontkowski et al. 1997 [39]	AO/OTA (dist. tibia)	0.49-0.58	-
Yacoubian et al. 2002 [43]	Müller-AO, Schatzker (tibial plateau)	0.68 (x-ray)	-
		0.73 (+ CT)	
		0.85 (+ MRI)	
			
**Other**			
Ward et al. 1997 [42]	Severin (hip)	0.16	0.34
McAdams et al. 2002 [24]	Scapular neck	0.30 (weighted) - 0.81	0.49 (weighted)
			
***Li-La***	***All long bones and localisations***	***0.71***	***0.78-0.93***

The optional fifth digit, which indicates a tolerable or non-tolerable dislocation, resulted in good interobserver agreement (κ = 0.83) if the non-displaced and the displaced but tolerable fractures were interpreted as one and the same class. The definition of displaced but tolerable and displaced and not tolerable fractures is currently based on the knowledge in the literature and enhances the clinical relevance substantially. In such a simplified mode, the fifth digit could be used in further studies for evaluating guidance for treatment.

It has been suggested that a useful classification system must be hierarchical to offer guidance in determining the optimal treatment method and to indicate the prognosis for a particular condition [34,38-40]. In contrast to adult classifications, a hierarchical order for the paediatric fracture types (by severity, diagnostic or therapeutic management, or prognosis) is not possible or advisable because these parameters are influenced by many different factors. The injury pattern of children is stereotypical and seems to be much more dependent on the maturation stage of the physis than on the injury mechanism. This is why complicated articular fractures, as seen in adults, are not found in children as long as the epiphyseal plate is still wide open. Besides factors such as fracture localisation and extent of displacement, the choice of treatment is mainly influenced by the patient's age, since the prognosis for growth depends on this. It is also influenced by the growth plates and their maturity. Hence only a classification without hierarchies, which follows the neutral aspects of localisation and morphology, is useful in describing fractures in children. These non-hierarchical classifications mostly describe specific fractures of single localisations [36,41,42].

To our knowledge only one classification system of paediatric long bone fractures has been proposed to date. Its development and evaluation by the AO Paediatric expert group [24,25] proceeded at approximately the same time as the one presented in this paper. Hence, there are some similarities, but there are also important differences:

• The main distinction concerns the precise separation of the intraarticular fractures from the fractures not involving the articular surface. The AO system classifies separation of the physis as an articular fracture. However, a separation of the physis with or without metaphyseal wedge, generally known as Salter I and II fractures, does not involve the articular surface. These fractures are considered as the most peripheral shaft fractures of long bones. Thus, they have a different prognosis and need to be treated differently. In our opinion this aspect must be clearly considered in a paediatric classification system, which will ultimately be used to develop treatment guidelines and prognostic predictors.

• It has been shown that the simpler the fracture classification, the better its reliability [10,12,15,43]. For these reasons we tried to simplify our classification system to the necessary minimum. All infrequent lesions (0-1% of all fractures) were subsumed in one category. The only exception was the articular fracture of the distal humerus, due to its importance. In contrast, the AO classification [24,25] includes different exceptions and additional codes, e.g. for supracondylar fractures, and fractures of the radial head or the proximal femur.

## Conclusions

In conclusion, we have developed a paediatric classification system for fractures of the long bones, which has been shown to have good reliability. This classification system also accommodates determination of clinical consequences and hence surpasses the simple description and definition of fractures. We therefore propose use of this classification system in future prospective studies including those examining the relevance of therapeutic measures. The latter should include evaluation of the minimum necessary diagnostic and therapeutic procedures leading to an optimum outcome.

## Competing interests

The authors declare that they have no competing interests.

## Authors' contributions

DS is the principal investigator and organizer of the rating sessions. She drafted the manuscript in collaboration with CR who was also responsible for organization of the Li-La multicenter studies. RK and LvL are the main drivers behind the development of the classification system on behalf of the Li-La group. IM contributed with clinical and methodological expertise and hosted all rating sessions. MK applied the classification system in his hospital in a separate one-year prospective study and helped further developing and refining it. DD conducted all statistical analyses. All authors read and approved the final manuscript.

## Pre-publication history

The pre-publication history for this paper can be accessed here:

http://www.biomedcentral.com/1471-2474/12/89/prepub
